# Which doctors and with what problems contact a specialist service for doctors? A cross sectional investigation

**DOI:** 10.1186/1741-7015-5-26

**Published:** 2007-08-28

**Authors:** Antony I Garelick, Samantha R Gross, Irene Richardson, Matthias von der Tann, Julia Bland, Rob Hale

**Affiliations:** 1 MedNet Service for Doctors, Tavistock & Portman NHS Trust, 120 Belsize Lane, London NW3 5BA, UK.; 2 National Addiction Centre, Institute of Psychiatry, King's College London, DeCrespigny Park, Denmark Hill, London SE5 8AF, UK.; 3 Tavistock & Portman NHS Trust, 120 Belsize Lane, London NW3 5BA, UK.; 4 MedNet Service for Doctors, Maudsley Hospital, Denmark Hill, London SE5 8AZ, UK.

## Abstract

**Background:**

In the United Kingdom, specialist treatment and intervention services for doctors are underdeveloped. The MedNet programme, created in 1997 and funded by the London Deanery, aims to fill this gap by providing a self-referral, face-to-face, psychotherapeutic assessment service for doctors in London and South-East England. MedNet was designed to be a low-threshold service, targeting doctors without formal psychiatric problems. The aim of this study was to delineate the characteristics of doctors utilising the service, to describe their psychological morbidity, and to determine if early intervention is achieved.

**Methods:**

A cross-sectional study including all consecutive self-referred doctors (n = 121, 50% male) presenting in 2002–2004 was conducted. Measures included standardised and bespoke questionnaires both self-report and clinician completed. The multi-dimensional evaluation included: demographics, CORE (CORE-OM, CORE-Workplace and CORE-A) an instrument designed to evaluate the psychological difficulties of patients referred to outpatient services, Brief Symptom Inventory to quantify caseness and formal psychiatric illness, and Maslach Burnout Inventory.

**Results:**

The most prevalent presenting problems included depression, anxiety, interpersonal, self-esteem and work-related issues. However, only 9% of the cohort were identified as severely distressed psychiatrically using this measure. In approximately 50% of the sample, problems first presented in the preceding year. About 25% were on sick leave at the time of consultation, while 50% took little or no leave in the prior 12 months. A total of 42% were considered to be at some risk of suicide, with more than 25% considered to have a moderate to severe risk. There were no significant gender differences in type of morbidity, severity or days off sick.

**Conclusion:**

Doctors displayed high levels of distress as reflected in the significant proportion of those who were at some risk of suicide; however, low rates of severe psychiatric illness were detected. These findings suggest that MedNet clients represent both ends of the spectrum of severity, enabling early clinical engagement for a significant proportion of cases that is of importance both in terms of personal health and protecting patient care, and providing a timely intervention for those who are at risk, a group for whom rapid intervention services are in need and an area that requires further investigation in the UK.

## Background

The prevalence of psychological morbidity in practicing doctors has been estimated at 25% [1,2]. As a professional group, doctors are highly stressed [3-5], which is a situation that has been progressively increasing over time [6], and may be more susceptible to depression [7,8], burnout and anxiety; However, robust comparative research on this topic is limited [9]. Women doctors have consistently been shown to have a higher risk of suicide than their male counterparts, and their suicide rates may be higher than for the general population [10]. However, rates of attempted suicide may be lower for doctors overall compared to the general population [11,12]. Despite these factors, doctors are notoriously reluctant to use health services, often citing profound concerns about confidentiality and stigma [13-15].

In the UK, treatment services specifically targeted for doctors are underdeveloped and little is know about the doctors that attend these services [16] or the consequences of their problems on either personal health or clinical practice in the long-term. While doctors may access telephone counselling or mentoring through their employer, membership society and some of the Royal Colleges, these programmes do not offer face to face contact in a specialised designated clinic for doctors. This study, the first of its type, has investigated by direct clinical evaluation a cohort of doctors attending a specialist psychotherapeutic service for doctors in distress.

The aim of this study was to provide a comprehensive description of doctors seeking help at one service for psychological difficulties, based not only on standardised questionnaires, but also on clinical interviews and clinician ratings.

## Methods

### The service

MedNet was established in 1997 by the London Deanery, which is now responsible for all postgraduate education in the London area. The MedNet service also covers an area of southern England (Kent, Surrey and Sussex Deanery). It is a self-referral service available to all doctors, not only those in training. The Tavistock Clinic hosts the MedNet service in North London; in South London it is hosted at the Maudsley Hospital. The service is staffed by three Consultant Psychiatrists in Psychotherapy on a sessional basis and a full-time secretary, and offers confidential brief assessments and longer-term psychological support as required. The consultations take account of the practical and cultural aspects of medical practice in the UK and explore psychological health, work-life balance and career issues. The aim of the service is to provide a therapeutic opportunity for doctors to identify and clarify the nature of their problems and how they interact with their professional and personal lives.

### The sample

All doctors who contacted the service between February 2002 and February 2004 were eligible for inclusion (n = 123). Of these, 98.4% (n = 121) consented to take part. Complete data was obtained for 107 clients, and partial data was collected for 14.

### Measures

Data collection included standardised and bespoke questionnaires, utilising both self-report (prior to index appointment) and clinician-completed assessments (during index appointment). Bespoke questions included information on demographics, personal and family health history, education and professional training.

Information concerning levels of risk and the range, intensity and duration of problems was collected using the clinician-rated CORE Assessment (CORE-A) and the CORE Workplace Therapy Assessment Form (CORE-Workplace) [17]. Both of these tools provide for a broad picture of mental state and are not time limited.

Distress symptoms in the preceding week and standardised assessment of severity of presentation were captured by the CORE Outcome Measure (CORE-OM) and the Brief Symptom Inventory (BSI). The CORE-OM [18], which contains 34-items using a 5-point Likert response ('not at all' to 'most or all of the time'), provides scores for subjective wellbeing, commonly experienced problems or symptoms, life/social functioning and risk to self and others was also completed. Standardised scores were used to determine clinical cut points on this measure as provided by the distributor [18]. The Brief Symptom Inventory (BSI) [19], a 53-question, 5-point Likert scale (responses ranging from 'not at all' to 'extremely') self-rated questionnaire that targets symptoms in nine areas, was used to quantify 'caseness'. Caseness implies the criterion for requiring a formal mental health assessment and was computed using adult gender matched non-patient norms. Adult psychiatric outpatient norms for the BSI and the Global Severity Index (GSI) as a component of the BSI were used to measure clients' degree of symptomatic distress [19].

Work-related distress was evaluated through the three dimensions of the Maslach Burnout Inventory (MBI) [20]: 'emotional exhaustion', 'depersonalisation' and 'personal accomplishment', each of which is scored independently. The MBI is a 22-item questionnaire about job-related feelings, which uses a 7-point Likert scale to capture how often the individual has ever felt a certain way (responses 'never' to 'every day'). This questionnaire, like the CORE Workplace and CORE-A, is not as limited to point in time assessment.

### Data analysis

Statistical analyses were conducted in SPSS v. 15 and included descriptives, Chi-Squared tests for categorical measures, t-tests or Mann-Whitney U test for skewed data, as well as Pearson's correlation coefficient to identify relationships between scale data.

## Results

### Demographics

**Figure 1:**
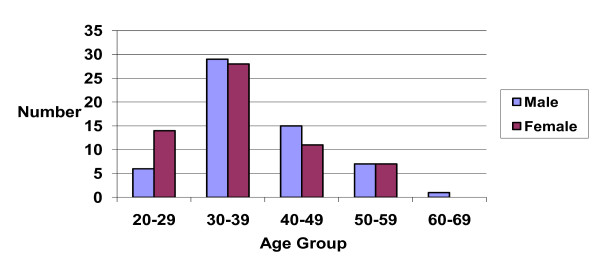
Age distribution.

An equal proportion of males (n = 61, 50%) and females (n = 60) presented to the service, with a mean age of 37 ± 8.6 years (range 24–64, Figure 1). The largest age group attending were 30–39 years (47%). There was no significant difference between the ages of male and female clients. The majority of clients were Caucasian (n = 79, 65%), with the largest minority group Asian (n = 24, 20%). Most were married (n = 68) and about 25% were single (n = 33). Approximately 33% had children.

**Figure 2:**
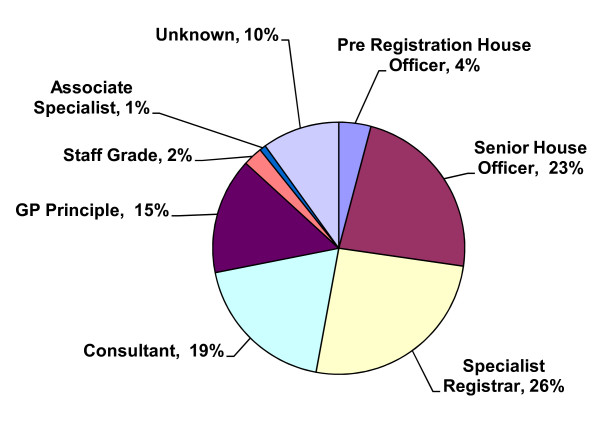
Training grade.

**Figure 3:**
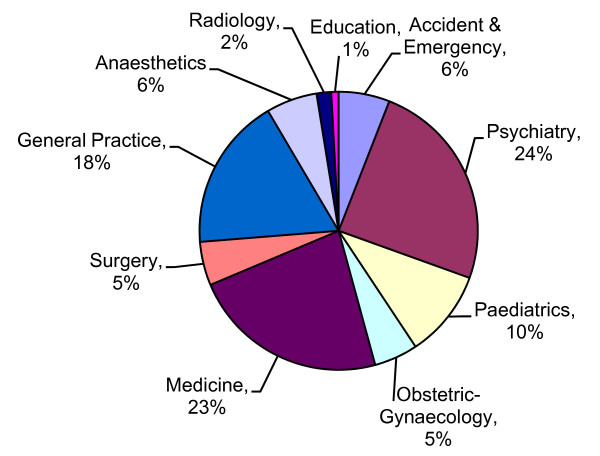
Medical speciality (n = 112).

Clients represented a variety of career grades (Figure 2), with specialist registrars the largest group represented (n = 31) followed closely by senior house officers (n = 28). A total of 23 consultants attended, of which nine were in post ≤ 5 years. There were no significant differences identified by gender and career grade. A total of 75% (n = 89) stated that they had passed postgraduate exams, and 88 had completed a higher training. The specialties of psychiatry (n = 29) and medicine (n = 27) were the largest groups represented (Figure 3), followed closely by those specialising in general practice (n = 21).

While all clients accessed the service via self-referral, 87 (72%) indicated that they were encouraged to do so by at least one individual. Nine were referred by multiple parties. These included their GP (n = 3), occupational health (n = 22), senior colleague (education) (n = 11), senior colleague (clinical) (n = 27) and/or their postgraduate deanery (n = 15), psychiatrist/psychologist/counsellor (n = 11), and a colleague, friend or partner (n = 7). For 10 clients referral route was not recorded. A total of 76 (63%) respondents indicated that they had seen their GP in the past 12 months. Approximately 50% (n = 58) had had a previous consultation with a psychiatrist.

### Presenting problems

Data from clinician completed CORE-A (Table 1) indicated that the most prevalent presenting problems were depression, anxiety/stress, interpersonal problems, self-esteem problems, and work-related problems. These were coded as moderate or severe in the majority of cases and for a significant minority of doctors, these problems had appeared in the preceding year.

**Table 1:** CORE-A, presenting problems rated by therapist

**Presenting Problems**	**N**	**With problem**		**Problem rated as moderate or severe**		**Problem arisen in past 12 months**	
		
		**%**	**n**	**n**	**%**	**n**	**%**
Depression	112	81.3	91	56	61.5	46	50.5
Anxiety	110	58.2	64	43	67.2	31	48.4
Interpersonal	111	45.9	51	32	62.7	11	21.6
Self esteem	112	38.4	43	24	55.8	14	32.6
Work/academic	111	32.4	36	21	58.3	16	44.4
Personality	111	24.3	27	10	37	0	37
Bereavement	111	15.3	17	11	64.7	8	47.1
Trauma	112	9.8	11	9	81.8	4	36.4
Addictions	112	5.4	6	3	50	1	16.7
Living/welfare	112	3.6	4	4	100	3	75
Psychosis	112	2.7	3	1	33.3	0	0
Physical	112	2.7	3	2	66.7	1	33.3
Other: sexual	112	2.7	3	1	33.3	-	-
Cognitive/learning	112	0.9	1	0	0	1	100
Eating disorder	112	0.9	1	1	100	1	100
Risk: suicide	110	41.8	46	12	26.1	-	-
Risk: self harm	110	22.7	25	8	32	-	-
Risk: harm to others	110	7.3	8	1	12.5	-	-
Risk: forensic/legal	110	0.9	1	0	0	-	-

According to therapist rating on the CORE-A, 42% were considered to be at some risk of suicide, with more than 25% (n = 12) considered to have a moderate to severe risk. A larger number of males (n = 27) than females (n = 19) were considered to be at risk, but this was not statistically significant. Eleven (9.1%, 3 male) clients had attempted suicide in the past, six on more than one occasion. In eight cases, an attempt had taken place in the 12 months preceding their contacting the service. Six of those who had attempted suicide in the past were considered to be at moderate to high suicidal risk at the time of presentation. None of those who had attempted suicide in the past were considered a risk for harm to others or a forensic risk.

Mean scores on the CORE-OM subscales at intake are detailed in Table 2. Clients scored worse than the general population but better than the clinical population (e.g. out-patients) on all sub-scales. Overall, 63% (n = 72) of those who completed the assessment scored above clinical cut-off (1.19 for males, 1.29 for females) on their CORE-OM total score. A similar proportion of males (61%) and females (64%) reached clinical cut-off as did a similar proportion of junior (66%) middle (54.5%) and senior (68.3%) grades. The only significant difference identified on the CORE-OM was that males had a lower score on the Wellbeing subscale than females (X¯
 MathType@MTEF@5@5@+=feaafiart1ev1aaatCvAUfKttLearuWrP9MDH5MBPbIqV92AaeXatLxBI9gBaebbnrfifHhDYfgasaacH8akY=wiFfYdH8Gipec8Eeeu0xXdbba9frFj0=OqFfea0dXdd9vqai=hGuQ8kuc9pgc9s8qqaq=dirpe0xb9q8qiLsFr0=vr0=vr0dc8meaabaqaciaacaGaaeqabaqabeGadaaakeaacuqGybawgaqeaaaa@2DFB@ = 1.77 vs X¯
 MathType@MTEF@5@5@+=feaafiart1ev1aaatCvAUfKttLearuWrP9MDH5MBPbIqV92AaeXatLxBI9gBaebbnrfifHhDYfgasaacH8akY=wiFfYdH8Gipec8Eeeu0xXdbba9frFj0=OqFfea0dXdd9vqai=hGuQ8kuc9pgc9s8qqaq=dirpe0xb9q8qiLsFr0=vr0=vr0dc8meaabaqaciaacaGaaeqabaqabeGadaaakeaacuqGybawgaqeaaaa@2DFB@ = 2.26 respectively, t = -2.8, p < 0.01).

**Table 2:** Scores on CORE-OM (n = 115) and MBI (n = 114) at intake

**Measure**	**Sub-scale**	**Score range**	**Pre-intervention**		**Above clinical cut-off**		Published norms*	
			**Mean**	**(SD)**	**n**	**(%)**	**Mean**	**(SD)**

CORE-OM	Functioning	0–3.75	1.41	0.76	-	-	0.85	0.65
	Problems	0–3.92	1.84	0.86	-	-	0.90	0.72
	Wellbeing	0–4.00	2.02	0.97	-	-	0.91	0.83
	Risk	0–3.00	0.32	0.48	-	-	0.20	0.45
	Total score	0–3.68	1.44	0.69	72	62.6	0.76	0.59
MBI	Emotional exhaustion	Jan-54	31.05	12.5	73	64	22.19	9.53
	Depersonalisation	0–30	8.8	6.95	44	38.6	7.12	5.22
	Personal accomplishment	Feb-46	34.32	8.09	45	39.5	36.53	7.34
	Burnout**	-	-	-	21	18.4	-	-

*Normative data adapted from [17] (norms for non-clinical sample) and [20] (norms for sample in medicine); **high emotional exhaustion, depersonalisation and low personal achievement.

The BSI (n = 114, Table 3) was used to assess clinical severity of presentation. Caseness was computed using two different methods. The first, by scoring over clinical cut-off on two or more sub-scales (a t-score of ≤ 63) (n = 93) and the second by scoring over clinical cut-off on the Global Severity Index (n = 87). When t-score was based on general population norms, 85.1% (n = 97) of clients could be defined as potential psychiatric cases (i.e. individuals requiring formal mental health assessment) by either of these methods. However, when t-score was calculated based on adult outpatient psychiatric norms, only 9% (n = 11 of 114) scored above cut-off, indicating that only a small proportion were severely psychiatrically distressed.

**Table 3:** Raw scores on the BSI at intake and comparisons with normative data (n = 114)

**Measure**	**Sub-scale**	**Range**	**MedNet pre-intervention**		Adult non-patient population published norms (US)*		**Adult psychiatric outpatient population published norms (US)^**		**Above clinical cut-off: non-patient**		**Above clinical cut off: psychiatric outpatient**	
			**Mean**	**(SD)**	**Mean**	**(SD)**	**Mean**	**(SD)**	**n**	**(%)**	**n**	**(%)**

**BSI**	Somatization	0–3.57	0.44	0.62	0.29	0.40	0.83	0.79	20	17.5	5	4.4
	Obsessive-compulsive	0–4	1.42	0.95	0.43	0.48	1.57	1.00	71	62.3	9	7.9
	Interpersonal sensitivity	0–4	1.36	1.01	0.32	0.48	1.58	1.05	73	64.0	8	7
	Depression	0–4	1.48	0.98	0.28	0.46	1.80	1.08	76	66.7	6	5.3
	Anxiety	0–4	1.13	0.94	0.35	0.45	1.70	1.00	54	47.4	5	4.4
	Hostility	0–3.6	0.74	0.75	0.35	0.42	1.16	0.93	35	30.7	6	5.3
	Phobic anxiety	0–3.8	0.45	0.74	0.17	0.36	0.86	0.88	33	28.9	7	6.1
	Paranoid ideation	0–3.4	0.92	0.86	0.34	0.45	1.14	0.95	49	43.0	7	6.1
	Psychoticism	0–3	0.81	0.67	0.15	0.30	1.19	0.87	70	61.4	4	3.5
	Global Severity index	0–3.51	0.98	0.65	0.30	0.31	1.32	0.72	87	71.9	5	4.4
	Positive Symptom Distress index	0–3.58	26.33	10.53	11.45	9.20	30.80	11.63	-	-	-	-
	Positive Symptom total	0–53	1.83	0.57	1.29	0.40	2.14	0.61	-	-	-	-

* From [19].

On the MBI (n = 114, Table 2), about 66%of clients presented with high levels of emotional exhaustion (score ≤ 27), 39% (score ≤ 10) with a high level of depersonalisation, and 40% (score ≥ 33) were classified with a low level of personal accomplishment. Males had significantly higher scores on the personal accomplishment construct than females (X¯
 MathType@MTEF@5@5@+=feaafiart1ev1aaatCvAUfKttLearuWrP9MDH5MBPbIqV92AaeXatLxBI9gBaebbnrfifHhDYfgasaacH8akY=wiFfYdH8Gipec8Eeeu0xXdbba9frFj0=OqFfea0dXdd9vqai=hGuQ8kuc9pgc9s8qqaq=dirpe0xb9q8qiLsFr0=vr0=vr0dc8meaabaqaciaacaGaaeqabaqabeGadaaakeaacuqGybawgaqeaaaa@2DFB@ = 36 vs X¯
 MathType@MTEF@5@5@+=feaafiart1ev1aaatCvAUfKttLearuWrP9MDH5MBPbIqV92AaeXatLxBI9gBaebbnrfifHhDYfgasaacH8akY=wiFfYdH8Gipec8Eeeu0xXdbba9frFj0=OqFfea0dXdd9vqai=hGuQ8kuc9pgc9s8qqaq=dirpe0xb9q8qiLsFr0=vr0=vr0dc8meaabaqaciaacaGaaeqabaqabeGadaaakeaacuqGybawgaqeaaaa@2DFB@ = 32.7, t = 2.2, p < 0.05), however there was no significant difference between genders on reaching cut-off for burnout on this domain. Using the traditional method for interpretation of this scale, where 'burnout' equals high depersonalisation and emotional exhaustion and low personal achievement, 21 clients (18.4%) reached this threshold. If one were to exclude the personal achievement construct, as recent authors have performed based on its' independence [8], the proportion of clients reaching classification of burnout increases to approximately 33% (n = 36).

### Work-related difficulties

More than 25% of clients (n = 31) had not taken any sick leave during the year prior accessing the service (11 did not respond). Of the 79 clients who had been on sick leave (X¯
 MathType@MTEF@5@5@+=feaafiart1ev1aaatCvAUfKttLearuWrP9MDH5MBPbIqV92AaeXatLxBI9gBaebbnrfifHhDYfgasaacH8akY=wiFfYdH8Gipec8Eeeu0xXdbba9frFj0=OqFfea0dXdd9vqai=hGuQ8kuc9pgc9s8qqaq=dirpe0xb9q8qiLsFr0=vr0=vr0dc8meaabaqaciaacaGaaeqabaqabeGadaaakeaacuqGybawgaqeaaaa@2DFB@ = 46 ± 74 days, median = 14), 31 (41%) had been on sick leave for a maximum of 7 days. In contrast, 34 clients had been on sick leave between 21 days (more than 1 working month) and 1 year (X¯
 MathType@MTEF@5@5@+=feaafiart1ev1aaatCvAUfKttLearuWrP9MDH5MBPbIqV92AaeXatLxBI9gBaebbnrfifHhDYfgasaacH8akY=wiFfYdH8Gipec8Eeeu0xXdbba9frFj0=OqFfea0dXdd9vqai=hGuQ8kuc9pgc9s8qqaq=dirpe0xb9q8qiLsFr0=vr0=vr0dc8meaabaqaciaacaGaaeqabaqabeGadaaakeaacuqGybawgaqeaaaa@2DFB@ = 98.32 ± 88.7 days, median = 65). Where recorded, reasons for prolonged sick leave included psychological problems (n = 19, primarily depression), both psychological and physical problems (n = 6), and physical problems only (n = 9, including cold/virus, asthma and surgery), four of whom required hospitalisation. There were no significant differences in number of sick days by gender or by career grade.

More than 75% of clients (n = 95) were dealing with work-related difficulties at the time of presentation, however only 36 clients were identified to have work issues as one of their primary presenting problems. Work issues were coded moderate or severe in 50 to 85% of cases, the majority appearing in the 12 months prior to the assessment (Table 4). Work functioning was considered by therapists normal or satisfactory in 55% (n = 59) of clients, impaired or severely impaired in 15% (n = 16). A total of 27%(n = 29) were on sick leave at the time of assessment. Four of those whose work functioning was impaired (n = 13) and all of those with severe impairment (n = 3) had been on sick leave in excess of 20 days in the preceding year. There were no significant differences identified by gender and work-related difficulties. Many (54%, n = 65) had work-related difficulties spanning multiple domains. Eight had or were presently undergoing formal proceedings at work (7 male), and two were suspended for clinical performance issues.

**Table 4:** CORE-WORKPLACE therapist assessment, n = 108

**Domain**	**With problem**		**Moderate/severe**		**Problem appeared in last 12 months,**	
	**n**	**(%)**	**n**	**(%)**	**n**	**(%)**

Change of job	29	26.9	21	72.4	23	79.3
Workload	48	44.4	37	77.1	19	39.6
Work conditions	20	18.5	17	85	12	60
Work relationships	32	29.6	22	68.8	12	37.5
Bullying	12	11.1	6	50	7	58.3
Traumatic event	5	4.6	4	80	5	100
Violence	1	0.9	1	100	1	100
Work-related health	10	9.3	7	70	6	60
Career issues	29	26.9	16	55.2	12	41.4
Organisational issues	8	7.4	6	75	4	50
Formal proceedings	8	7.4	5	62.5	6	75
Other	16	14.8	12	75	14	87.5

Days of sickness absence taken in the preceding year were significantly correlated with scores on each of the standardised psychological assessments used and the majority of their subscales, excluding anxiety and hostility on the BSI, problems and wellbeing on the CORE-OM and depersonalisation on the MBI. In addition, a larger proportion of those who had taken ≤ 21 days sickness absence in the preceding year (n = 34) met the classification for burnout on the MBI (χ^2 ^= 5.1, p < 0.05), or scored higher than normal population on the BSI (χ^2 ^= 4.82, p < 0.05) but there was no significant relationship between reaching clinical cut-off on the CORE-OM or the BSI for adult psychiatric outpatient norms and taking ≤ 21 days sick leave. Doctors who had taken extended leave were rated with consistently higher mean workplace problem severity than those who had taken less or no time off, however this difference was only significant for the therapist rating of workload problems (X¯
 MathType@MTEF@5@5@+=feaafiart1ev1aaatCvAUfKttLearuWrP9MDH5MBPbIqV92AaeXatLxBI9gBaebbnrfifHhDYfgasaacH8akY=wiFfYdH8Gipec8Eeeu0xXdbba9frFj0=OqFfea0dXdd9vqai=hGuQ8kuc9pgc9s8qqaq=dirpe0xb9q8qiLsFr0=vr0=vr0dc8meaabaqaciaacaGaaeqabaqabeGadaaakeaacuqGybawgaqeaaaa@2DFB@ = 3.6 vs X¯
 MathType@MTEF@5@5@+=feaafiart1ev1aaatCvAUfKttLearuWrP9MDH5MBPbIqV92AaeXatLxBI9gBaebbnrfifHhDYfgasaacH8akY=wiFfYdH8Gipec8Eeeu0xXdbba9frFj0=OqFfea0dXdd9vqai=hGuQ8kuc9pgc9s8qqaq=dirpe0xb9q8qiLsFr0=vr0=vr0dc8meaabaqaciaacaGaaeqabaqabeGadaaakeaacuqGybawgaqeaaaa@2DFB@ = 2.8, t = -3.4, p = 0.001). About 33% of those who reached classification for burnout were also classified with difficulties associated with job change on the CORE-Workplace (n = 7, χ^2 ^= 3.8, p = 0.05); there were no other significant relationships between these measures.

### Group at risk

Of the 11 cases that had attempted suicide in the past, eight scored above clinical cut-off on the CORE-OM, two were classified with burnout according to the MBI, one who was off work for the entirety of the preceding year and the other took less than 20 days off. All were cases according to the BSI and two scored above adult psychiatric outpatient norms. This group had a higher number of sick days in the preceding year (X¯
 MathType@MTEF@5@5@+=feaafiart1ev1aaatCvAUfKttLearuWrP9MDH5MBPbIqV92AaeXatLxBI9gBaebbnrfifHhDYfgasaacH8akY=wiFfYdH8Gipec8Eeeu0xXdbba9frFj0=OqFfea0dXdd9vqai=hGuQ8kuc9pgc9s8qqaq=dirpe0xb9q8qiLsFr0=vr0=vr0dc8meaabaqaciaacaGaaeqabaqabeGadaaakeaacuqGybawgaqeaaaa@2DFB@ = 115 vs X¯
 MathType@MTEF@5@5@+=feaafiart1ev1aaatCvAUfKttLearuWrP9MDH5MBPbIqV92AaeXatLxBI9gBaebbnrfifHhDYfgasaacH8akY=wiFfYdH8Gipec8Eeeu0xXdbba9frFj0=OqFfea0dXdd9vqai=hGuQ8kuc9pgc9s8qqaq=dirpe0xb9q8qiLsFr0=vr0=vr0dc8meaabaqaciaacaGaaeqabaqabeGadaaakeaacuqGybawgaqeaaaa@2DFB@ = 55, z = 3.27, p < 0.001) although three had taken less than 1 month leave. Nine of this group had previously consulted a psychiatrist. Similarly, of the 12 who were classed with moderate or severe suicidal risk at the time of presentation, two scored above outpatient psychiatric norms (one had attempted suicide in the past), and three had taken negligible time off sick.

Clients with any degree of risk for suicide, self-harm, harm to others and forensic/legal harm by therapists on the CORE-A had significantly higher scores on the CORE-OM risk subscale (X¯
 MathType@MTEF@5@5@+=feaafiart1ev1aaatCvAUfKttLearuWrP9MDH5MBPbIqV92AaeXatLxBI9gBaebbnrfifHhDYfgasaacH8akY=wiFfYdH8Gipec8Eeeu0xXdbba9frFj0=OqFfea0dXdd9vqai=hGuQ8kuc9pgc9s8qqaq=dirpe0xb9q8qiLsFr0=vr0=vr0dc8meaabaqaciaacaGaaeqabaqabeGadaaakeaacuqGybawgaqeaaaa@2DFB@ = 0.44 vs X¯
 MathType@MTEF@5@5@+=feaafiart1ev1aaatCvAUfKttLearuWrP9MDH5MBPbIqV92AaeXatLxBI9gBaebbnrfifHhDYfgasaacH8akY=wiFfYdH8Gipec8Eeeu0xXdbba9frFj0=OqFfea0dXdd9vqai=hGuQ8kuc9pgc9s8qqaq=dirpe0xb9q8qiLsFr0=vr0=vr0dc8meaabaqaciaacaGaaeqabaqabeGadaaakeaacuqGybawgaqeaaaa@2DFB@ = 0.2; t = -2.7, p < 0.01), but remained below the published clinical sample mean of 0.63 [18]. This group also had a higher number of days off sick in the preceding year (X¯
 MathType@MTEF@5@5@+=feaafiart1ev1aaatCvAUfKttLearuWrP9MDH5MBPbIqV92AaeXatLxBI9gBaebbnrfifHhDYfgasaacH8akY=wiFfYdH8Gipec8Eeeu0xXdbba9frFj0=OqFfea0dXdd9vqai=hGuQ8kuc9pgc9s8qqaq=dirpe0xb9q8qiLsFr0=vr0=vr0dc8meaabaqaciaacaGaaeqabaqabeGadaaakeaacuqGybawgaqeaaaa@2DFB@ = 43 vs X¯
 MathType@MTEF@5@5@+=feaafiart1ev1aaatCvAUfKttLearuWrP9MDH5MBPbIqV92AaeXatLxBI9gBaebbnrfifHhDYfgasaacH8akY=wiFfYdH8Gipec8Eeeu0xXdbba9frFj0=OqFfea0dXdd9vqai=hGuQ8kuc9pgc9s8qqaq=dirpe0xb9q8qiLsFr0=vr0=vr0dc8meaabaqaciaacaGaaeqabaqabeGadaaakeaacuqGybawgaqeaaaa@2DFB@ = 58, z = 2.6, p = 0.01).

Clients who were classified severely distressed on the BSI had significantly higher scores on the CORE-OM total score (X¯
 MathType@MTEF@5@5@+=feaafiart1ev1aaatCvAUfKttLearuWrP9MDH5MBPbIqV92AaeXatLxBI9gBaebbnrfifHhDYfgasaacH8akY=wiFfYdH8Gipec8Eeeu0xXdbba9frFj0=OqFfea0dXdd9vqai=hGuQ8kuc9pgc9s8qqaq=dirpe0xb9q8qiLsFr0=vr0=vr0dc8meaabaqaciaacaGaaeqabaqabeGadaaakeaacuqGybawgaqeaaaa@2DFB@ = 2.4 vs X¯
 MathType@MTEF@5@5@+=feaafiart1ev1aaatCvAUfKttLearuWrP9MDH5MBPbIqV92AaeXatLxBI9gBaebbnrfifHhDYfgasaacH8akY=wiFfYdH8Gipec8Eeeu0xXdbba9frFj0=OqFfea0dXdd9vqai=hGuQ8kuc9pgc9s8qqaq=dirpe0xb9q8qiLsFr0=vr0=vr0dc8meaabaqaciaacaGaaeqabaqabeGadaaakeaacuqGybawgaqeaaaa@2DFB@ = 1.4, t = -5.36, p < 0.001) and on all subscales including depression, and on the MBI had a significantly higher score on the emotional exhaustion subscale (t = 2.07, p < 0.05) and a lower score on the personal accomplishment subscale (t = 4.04, p < 0.001). While they also had a significantly higher number of days off sick (z = 2.4, p < 0.05), two had taken negligible time off, with a further two taking less than 1 month off.

## Discussion

MedNet was established in order to assist doctors with problems who had hitherto not sought formal help. This service was thus not designed primarily for those known to established psychiatric illness. It was assumed that those with established psychological difficulties/psychiatric illness would already be in treatment (this includes doctors with substance misuse).

The strength of this study is that it describes in detail for the first time an under-researched group in the actual clinical setting. Furthermore, a strict assessment protocol was adhered to, involving the use of reliable and valid psychometric instruments. Nevertheless, the study presents a number of limitations in so far as it has no control group and clients referred themselves to the service (although they were often strongly encouraged to do so).

An almost equal number of male and female doctors referred themselves, and there was no difference in the degree of morbidity. As accurate baseline numbers for doctors and trainees within the regions covered by the MedNet service over the study period are not available, a measurement of the representativeness of our sample with regard to gender can not be made. However, the absence of differences in degree of morbidity by gender does not confirm previous work, which has found increased morbidity in female doctors, most often measured by the GHQ [1,2].

The largest group of doctors to attend were aged 30–39 years, who were specialist registrars coming to the end of their training and thus looking for consultant posts, there were also a significant number of newly-appointed consultants. This data lends support to the view that one of the most stressful times for registered doctors is the transition from trainee to full responsibility as either a consultant or principal general practitioner

Data from self-report measures show that 63% of the entire cohort of doctors seen had significant morbidity as measured by the CORE-OM and 85.1% reached caseness on the BSI, however only 9% scored above adult outpatient psychiatric norms indicating that only a small number were severely psychiatrically distressed. In terms of suicidal risk, 42% were considered to be at risk by the therapists. The BSI was able to reflect the level of general morbidity in this population, but was limited in being able to identify the specific population of suicidal doctors as identified clinically by the CORE-A.

A survey of newly qualified doctors in Norway found that 14% of doctors had seriously thought about or planned suicide in the preceding year [21]. In our sample, 11 had attempted suicide, eight in the preceding 12 months. This is well above the annual rate for attempted suicide in the general population, which is 0.5% per annum [11], with the doctors attending the service showing at least a 13-fold difference compared to the general population. Closer scrutiny of the higher risk group showed that only two were severely distressed according to the BSI. Three had negligible time off sick. The lack of a clear pattern in this special group, some working whilst suicidal, illustrates the difficulty in identifying such problems unless the individual is motivated to seek help.

A total of 28% of the cohort had been on sick leave for more than 4 working weeks (≤ 21 days) in the preceding year, the majority with depression. This adds further evidence to the finding that the doctors who attend are extensively distressed with a small sub-group who are at significant personal risk; clients with a history of attempted suicide having a significantly higher risk of suicide [22]. The fact that 50% took little or no leave in the preceding 12 months suggests that MedNet clients represent both ends of the spectrum of severity.

The fact that 72% were encouraged to attend by medical colleagues indicates that their problems had become visible enough to engender concern. The vast majority had completed their postgraduate exams, indicating that academic performance was not compromised. Clinical performance in the study was judged by self-report and the clinical judgement derived from consultation with the doctors. No direct objective evidence was available. The adverse effect of stress and poor health on performance is well documented [23]. More than 75% of this cohort reported that they had work-related issues that had arisen within the preceding year; however the majority did not have serious performance problems (there is no rating for impairment for those off sick, which may skew the results to underreporting performance problems). Up to 33% reached the criteria for burnout and there were high levels of emotional exhaustion as measured by the MBI. This data in conjunction with our clinical impression shows that in general clinical performance is relatively preserved, even though the individual doctor is significantly distressed and that despite serious distress, clients' jobs had not yet been jeopardised. Many of them, despite high levels of emotional exhaustion, had maintained medium to high levels of personal accomplishment. This would support the view that doctors with such difficulties put all their available energy into maintaining their professional identity by working through their illness [24], with their exhaustion manifesting itself when off duty.

Approximately 25% of the doctors were on sick leave more than one working month; this represents a group that merits further study. The main finding that emerged from this study about this group is that although there is a trend to higher levels of morbidity, concerns about work and associated significant degrees of burnout were the most important determinants of sick leave over 20 days duration.

## Conclusion

In summary, this specialist service was successful in enabling doctors with considerable distress both personally and in the workplace, a small number being at significant risk, to receive help. Although the majority of the doctors in the study had made occasional contact with their GP in the preceding year, our clinical impression is that they had not engaged in more intensive help before making contact with our services. We suggest that many of these doctors would not have done so if a confidential self-referral service was not available and that the findings lend weight that such services do achieve earlier engagement in a significant proportion of doctors attending, enhancing the possibility of 'nipping problems in the bud' both personally and professionally.

## Competing interests

The author(s) declare that they have no competing interests.

## Authors' contributions

RH wrote the protocol and obtained funding for this audit supporting IR as research fellow. AIG acted as the investigator for the later stages of the study following RH's retirement. SRG conducted secondary data analysis for this paper and prepared the present manuscript with AIG. All authors contributed to and approved the final manuscript.

## Pre-publication history

The pre-publication history for this paper can be accessed here:


